# Artificial intelligence-driven diagnosis of autism spectrum disorder in children

**DOI:** 10.3389/fmed.2025.1723320

**Published:** 2025-12-08

**Authors:** Nizar Alsharif, Abdullah H. Al-Nefaie, Sultan Ahmad, Nesren S. Farhah

**Affiliations:** 1 King Salman Center for Disability Research, Riyadh, Saudi Arabia; 2 Department of Computer Engineering and Science, Al-baha University, Al-baha, Saudi Arabia; 3 Department of Quantitative Methods, School of Business, King Faisal University, Al-Ahsa, Saudi Arabia; 4 Department of Computer Science, College of Computer Engineering and Sciences, Prince Sattam Bin Abdulaziz University, Alkharj, Saudi Arabia; 5 School of Computer Science and Engineering, Lovely Professional University, Phagwara, India; 6 Department of Health Informatics, College of Health Science, Saudi Electronic University, Riyadh, Saudi Arabia

**Keywords:** autism spectrum disorder, artificial intelligence, explainable artificial intelligence, diagnosing, machine learning

## Abstract

**Introduction:**

Autism Spectrum Disorder (ASD) significantly impacts society by highlighting the need for inclusive education, healthcare, and employment systems that support neurodiversity. This challenges societal norms and promotes greater awareness, understanding, and acceptance, encouraging communities to become more inclusive and supportive of individuals with diverse abilities.

**Methods:**

The main novelty is to identify and explore the key factors affecting ASD in Saudi Arabia and Egypt, aiming to improve early diagnosis through Explainable Artificial Intelligence (XAI) techniques, specifically SHapley Additive exPlanations (SHAP), LIME (Local Interpretable Model-agnostic Explanations), and Permutation Feature Importance (PFI). The research primarily employed decision tree (DT) and K-Nearest Neighbors (KNN) models combined with explainable AI (XAI), leading to significant improvements in diagnostic accuracy. The system was applied to a real dataset collected from various locations across Saudi Arabia, which is publicly available on Kaggle. Additionally, another ASD dataset from the Data Science Bank repository, sourced from participants in North Cairo Governorate, Egypt, was used for testing the system. Before analysis, the data was validated by removing outliers, filling missing data, and confirming the relevance of selected features. The study aims to enhance early diagnosis through XAI methods, including SHAP, LIME, and PFI.

**Results:**

The results show that KNN, when combined with XAI, achieved a high accuracy of 97% on the Saudi Arabia dataset and 92% on the Egypt dataset.

**Discussion:**

This approach has proven to be effective in developing more accurate and straightforward AI models for ASD diagnosis. It demonstrates that integrating advanced AI techniques with practical clinical applications can significantly improve the healthcare system in Saudi Arabia, leading to earlier ASD detection, better-informed treatment plans, and ultimately, an improved quality of life for those affected.

## 1 Introduction

People with ASD are affected by this condition, impacting not only those who have it but also their families and the nation’s healthcare system. Raising a child with ASD is more costly than raising a healthy child because individuals with ASD need specialized therapy, medical care, and support in many areas of life, including education. This condition also creates financial burdens and worries for caregivers, as they must pay for behavioral, speech, and occupational therapies, as well as special educational programs that are not fully covered by insurance.

Moreover, caregivers may face significant challenges due to having to reduce their working hours and increase their availability for care. As a result, their financial stability could decline (1, 2). ASD is associated with higher public health costs, which are allocated to educational and social support systems. Social engagement and work life can also be negatively impacted and limited by societal stigma surrounding autistic individuals, leading to greater reliance on the economy. The rise in ASD diagnoses prompts us to address the socioeconomic challenges through legislation, special programs in education and employment, and improved access to affordable therapies and support services (3, 4). The World Health Organization (WHO) reported that 6.25 out of every 1,000 people worldwide are affected by ASD (5). Various efforts have been made to determine the prevalence of ASD in different countries.

Adak et al. (6) conducted a systematic review of 27 studies related to the prevalence of ASD. The results indicated an average prevalence of 9.19 per 1,000 individuals. The occurrence of ASD showed significant variation across countries, genders, socioeconomic statuses, geographic regions, continents, and assessment tools.

Studies show that early detection and therapy can help children with ASD improve their social, communication, and cognitive skills, emphasizing the vital role of screening for ASD, which is crucial for accessing help as soon as possible. Conversely, delayed diagnosis—often due to difficulty accessing specialists or more precise evaluation methods—highlights the importance of developmental stages, which are sometimes missed when intervention is needed. An ML-powered tool could address these issues by helping to achieve more objective and accurate ASD diagnoses. It may be challenging for clinicians to identify the early signs of ASD right away; however, ML models like deep learning (DL) can analyze large datasets simultaneously. Integrating ML algorithms into screening tools could enable systematic evaluations and real-time diagnostic suggestions. When combined with rule-based classifiers, ML makes it easier for clinicians to understand and interpret how decisions are made (6–9).

ML functions by combining the development of systems based on historical data with the creation of intelligent algorithms that perform specific tasks. It is used across various medical fields, such as classifying ASD (10, 11), identifying class imbalance in ASD data (12, 13), and diagnosing dementia (14). Like rule-based models, ML-based models can be explained and may incorporate rules (15). These models follow a structured approach to detect patterns that are understandable and easy to study. This makes them accessible. For example, a decision tree can analyze a child’s behavioral patterns and use specific criteria from the data to give a clear, step-by-step explanation for diagnosing ASD (16). Covering algorithms can assist clinicians in learning more about the traits used to evaluate ASD by analyzing large datasets and pinpointing relevant behavioral signs [as shown in a study by (17)]. In addition to their accuracy in screening for ASD, this interpretability enhances the strength and reliability of proposed diagnoses.

Even though more people around the world are interested in using AI to study and diagnose ASD, most of the research has focused on neural signals or medical imaging. There have not been many studies in the Arab world that use behavioral and social data from questionnaires.

In the Arab context, particularly in Saudi Arabia and Egypt, there is a significant lack of research utilizing AI to evaluate the responses of parents or educators for the early detection of autism indicators. Furthermore, most autism diagnostic tests were developed in different languages and cultures, which makes them less accurate in the Arab context. This study aims to fill this gap by collecting original Arabic data through local questionnaires and utilizing AI algorithms for analysis, with the goal of developing a model that enables the early and accurate identification of autism spectrum disorder, while taking into account the cultural specificities of Arab society.

### 1.1 Motivation

There are few studies on the prevalence and causes of ASD in Saudi Arabia and Egypt, despite it becoming a growing public health concern worldwide. To enhance healthcare planning, support early detection of ASD, and implement timely interventions, understanding the demographic and environmental factors affecting the disorder is essential. However, most recent ASD predictor studies either focus on specific regions or do not provide clear conclusions. The main goal of this study is to use XAI methods to address ASD in Saudi Arabian and Egyptian children. The aim is to identify demographic and environmental risk factors and develop models that are understandable and useful for clinicians, policymakers, and families.

### 1.2 Research question

1. What is important features that effective the children Saudi Arabia and Egypt?
2. How accurately can XAI algorithms with ML ASD outcomes transparently communicate the impact of recognized risk factors?

### 1.3 Objective of studies

1. To assess and analyze the prevalence of ASD among Arabic children and investigate regional disparities.
2. To identify and assess the impact of environmental and demographic risk factors related to ASD and their connection with symptom severity.
3. To create and assess XAI-based prediction models for ASD identification that guarantee interpretability and facilitate clinical and policy decision-making.

### 1.4 Contribution

- The primary contribution of this study is to analyze and diagnose ASD in Saudi Arabia and Egypt.
- AIX approach was applied to both datasets to improve the ML algorithm and to select the significant features that are effective for detecting and doing ASD at an early stage.
- The proposed method demonstrates that the DT and KNN models, when combined with the AIX approach, achieved 97% accuracy using KNN.
- Statistical analysis methods were employed to identify factors contributing to the increase in ASD and to investigate the relationship between these factors across different regions in Saudi Arabia and Egypt.
- We used the chi-square analysis method to find the *p*-values and determine the correction between the class and each feature in both datasets.

## 2 Related works

This section summarizes previous research in the area. Traditional autism ASD diagnoses rely only on standardized clinical testing, which necessitates extensive medical. In this area, we have discussed the latest scientific findings on ASD by using ML methods. Many researchers have tried to study ASD behaviors.

To identify autistic individuals, Vakadkar et al. (18) suggested ML models. The primary objective of this study was to determine the developmental windows of vulnerability to autism in children. They developed an automated approach to predict ASD, enabling quicker diagnoses than before. The dataset was used to apply several ML algorithms, including support vector machines (SVM), logistic regression (LR), KNN, naive Bayes (NB), and random forest (RF) classifiers. Based on Q-CHAT-10, this dataset contains 1,054 occurrences and 18 characteristics. For maximum precision, logistic regression was the preferred approach. One limitation of this method was the small number of factors and instances in the dataset.

Using the UCI database, Erkan et al. (19) conducted their research with three datasets: AQ-10-Adult, AQ-10-Adolescence, and AQ-10-Child. The study aimed to find a simpler method for early-stage ASD diagnosis. After applying ML algorithms to the datasets, the authors discovered that the RF Classifier outperformed SVM and KNN on several datasets. In this study, data were randomly sampled 100 times for each trial to evaluate the models. Early detection of ASD seems possible with a large dataset; however, diagnosis accuracy improves with a larger data sample. Thabtah et al. (20) developed ASDTests, a mobile app. To serve a diverse user base, the app offers tests in eleven different languages. Each age group—toddlers, children, teenagers, and adults—has its own specific module.

To validate the psychometric properties of the Q-CHAT questionnaire, Ruta et al. (21) used a clinical sample obtained from the Italian healthcare system. The Q-CHAT is a diagnostic tool specifically designed for autism; it does not apply to other neurological disorders. The study involved 350 children, including developing children (126), children without ASD (50), and autistic children (174). Additionally, they examined the statistical data related to these three groups. Compared to children with developmental delays and typically developing children, the autistic group had significantly higher Q-CHAT test scores. To assess how well the Q-CHAT questionnaire might identify autistic children, Tartarisco et al. (22) used ML algorithms. The sample was collected from three different regions in Italy by Ruta et al. (23). The Italian sample demonstrated that the Q-CHAT screening procedure is cross-culturally valid. According to their dataset, SVM was the most effective mML model.

Garbulowski et al. (24) used DT and SHAP to help identify different subtypes of ASD. The aim was to gain a deeper understanding of the unique traits of each subtype. The Autism Brain Imaging Data Exchange (ABIDE) dataset was utilized, which includes both brain imaging and behavioral data. The results showed significant differences among the various forms of ASD. The DT method performed reasonably well in identifying the subtypes, and the SHAP values clearly demonstrated the importance of each attribute by highlighting differences in brain connection patterns and behavioral features among the subtypes.

Andrade et al. (25) examined different ML approaches, including decision trees (DT) and support vector machines (SVMs), to develop a diagnostic procedure for ASD. Their method achieved over 92% accuracy in diagnoses, improving the reliability of condition prediction. Ruan et al. (26) explored more advanced data, such as video-based contrastive learning, and used decision trees to enhance diagnostic accuracy. Their approach combined DT and video-based contrastive learning to analyze and categorize behaviors relevant to ASD. Contrastive learning utilized video data to distinguish between behavioral patterns. The decision tree models proved highly effective in classifying behaviors associated with ASD using video datasets. Alwidian et al. (27) and Asgarnezhad et al. (28) investigated how classification methods, especially rule-based models, can support early intervention in individuals with ASD. Asgarnezhad et al. (28) used clinical and behavioral data from individuals with ASD to develop models employing techniques such as Decision Stump, DT, Gradient Boosted Trees, ID3, and CHAID, among others. They reported accuracies exceeding 90%. Alwidian et al. (27) employed DT, RD, SVM, K-NN, and NB to detect autism in toddlers. Their results showed that models built with rules generated by the RF classifier were the most accurate, achieving over 97.2% accuracy, outperforming existing methods like SVM and K-NN. Qureshi et al. (29) conducted research using the ABIDE dataset with a random forest (RF) approach. Their findings indicated that RF outperforms traditional classification methods, achieving accuracy above 92%. Since Parkinson’s disease (PD) is among the metal-related diseases, Bacanin et al. (30) used an LSTM model to predict PD. Ganggayah et al. (31) wrote a review of literature related to ASD using data science, analyzing challenges and limitations in detecting and predicting ASD. Samuele et al. (32) applied AI approaches to diagnose ASD to evaluate the robustness of AI models in identifying the condition. Aldhyani et al. (33) used a DL model to diagnose ASD with video data as the classification dataset. Alsharif et al. (34) employed an ML model to classify ASD using standard datasets. Altomi et al. (35) used a DL model to detect ASD based on facial images. Syriopoulou et al. (36) provided an in-depth look at the development of ASD diagnosis, tracing its evolution from psychoanalytic roots to the integration of advanced AI technology. Al-Nefaie et al. (37) used a DL model to detect and classify ASD using images. Jeon et al. (38) applied XAI techniques with an ML model for diagnosing ASD, using a standard dataset. It is noted that the model achieved 94%. Omar et al. (39) used XAI techniques to develop the ASD system; the authors used ID and RF algorithms. Alsuliman and Al-Baity (40) applied XAI techniques with bio-inspired algorithms for ASD detection. Ben-Sasson et al. (41) applied XAI techniques based on gradient boosting for early diagnosis of ASD. Abbas et al. (42) also used XAI techniques with AutoML algorithms for classifying ASD.

## 3 Methodology

**Figure 1:**
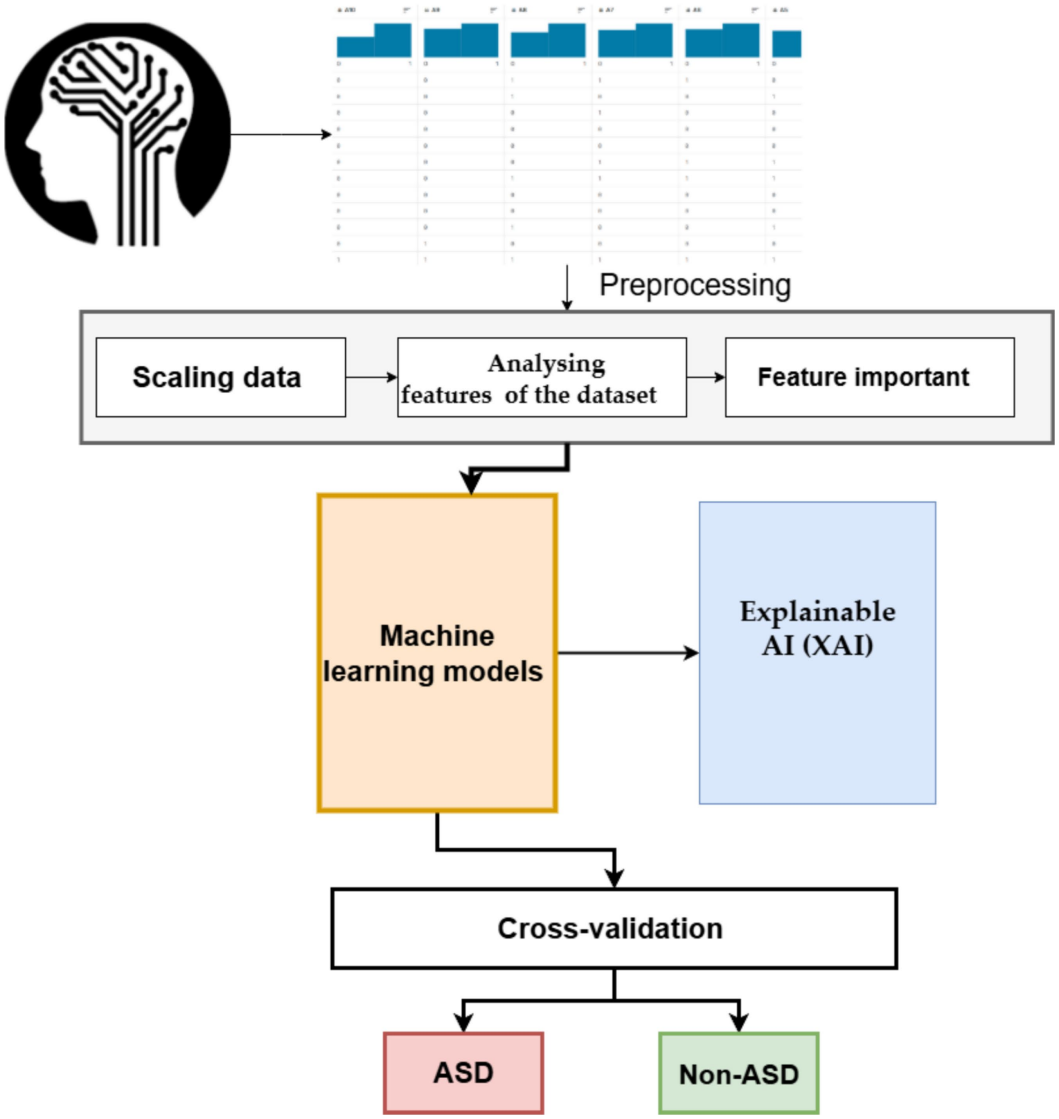
AI system for detecting ASD.

Figure 1 illustrates the structure and methodologies employed in utilizing DT and KNN models. XAI algorithms were used to identify the most irrelevant features of the dataset. The proposed procedural steps from preprocessing to model assessment emphasize the contribution of each step to achieving precise and interpretable ASD predictions.

### 3.1 Dataset

#### 3.1.1 Children of ASD in Saudi Arabia dataset

**Figure 2:**
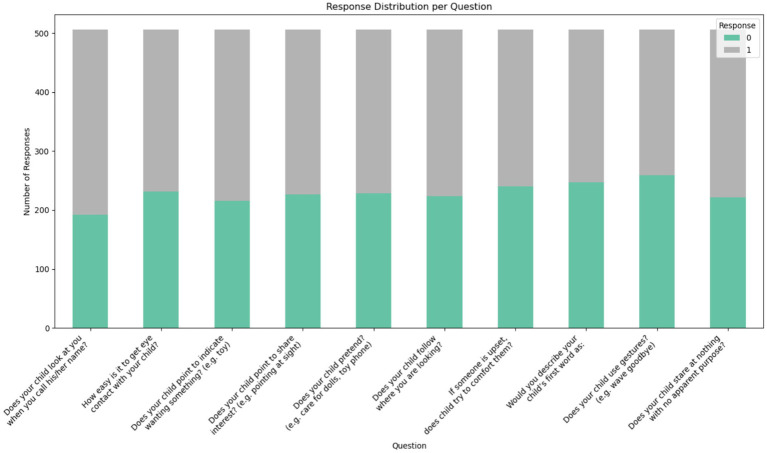
Features of the dataset.

**Figure 3:**
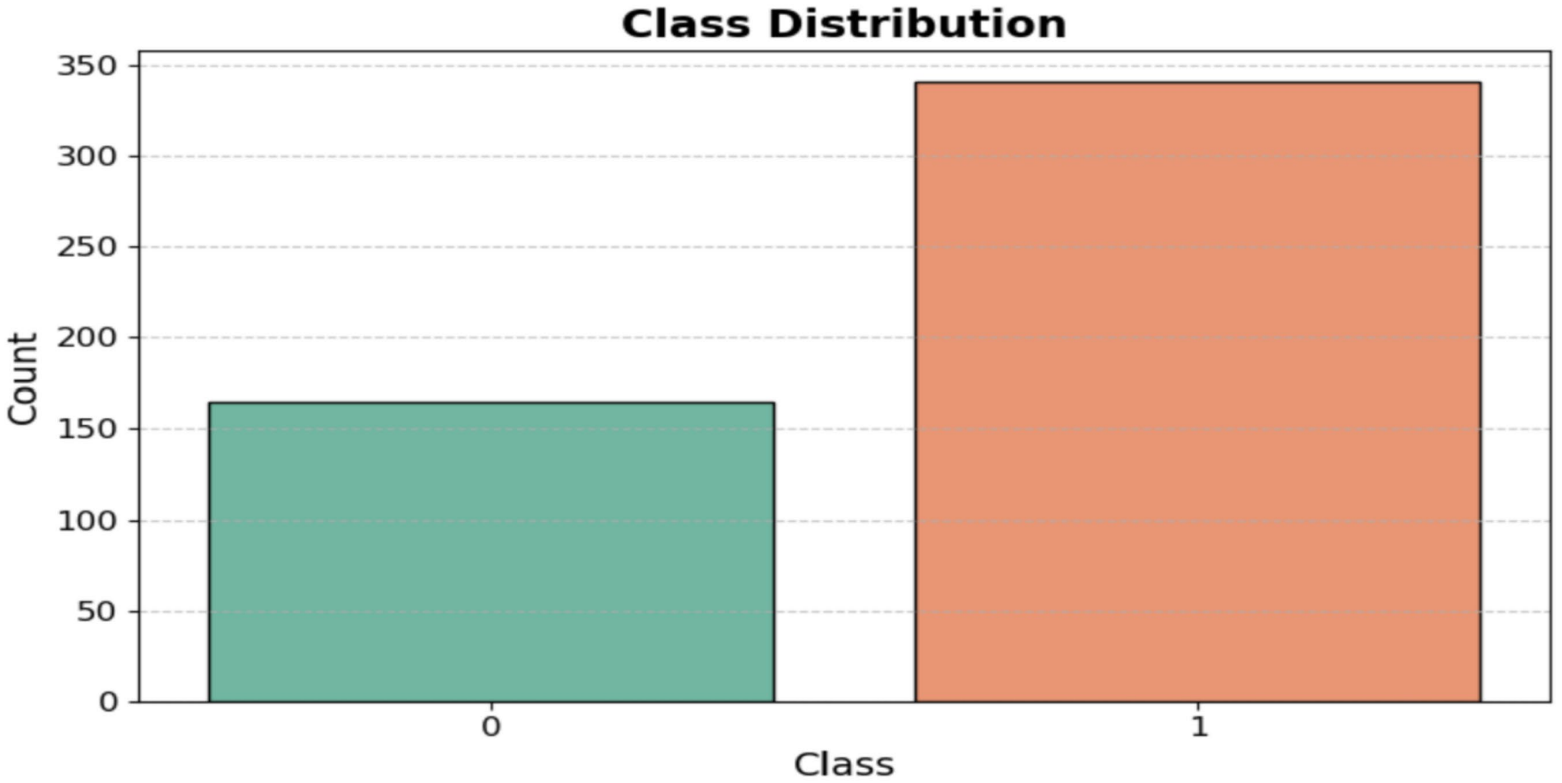
Class distribution.

The dataset was obtained from the Kaggle repository, which contains data on toddlers (ages 12 to 36 months) from various regions of Saudi Arabia. It includes both autistic and typically developing children. The data was collected using Google Forms, an online survey tool, to gather information such as age, gender, location, and family history of ASD. Figure 2 displays the questionnaire used in the dataset, highlighting the 10 most important questions related to the Q-CHAT test. The dataset categories are shown as [ASD and non-ASD] in Figure 3.

#### 3.1.2 Children’s ASD of the Egypt dataset

**Figure 4:**
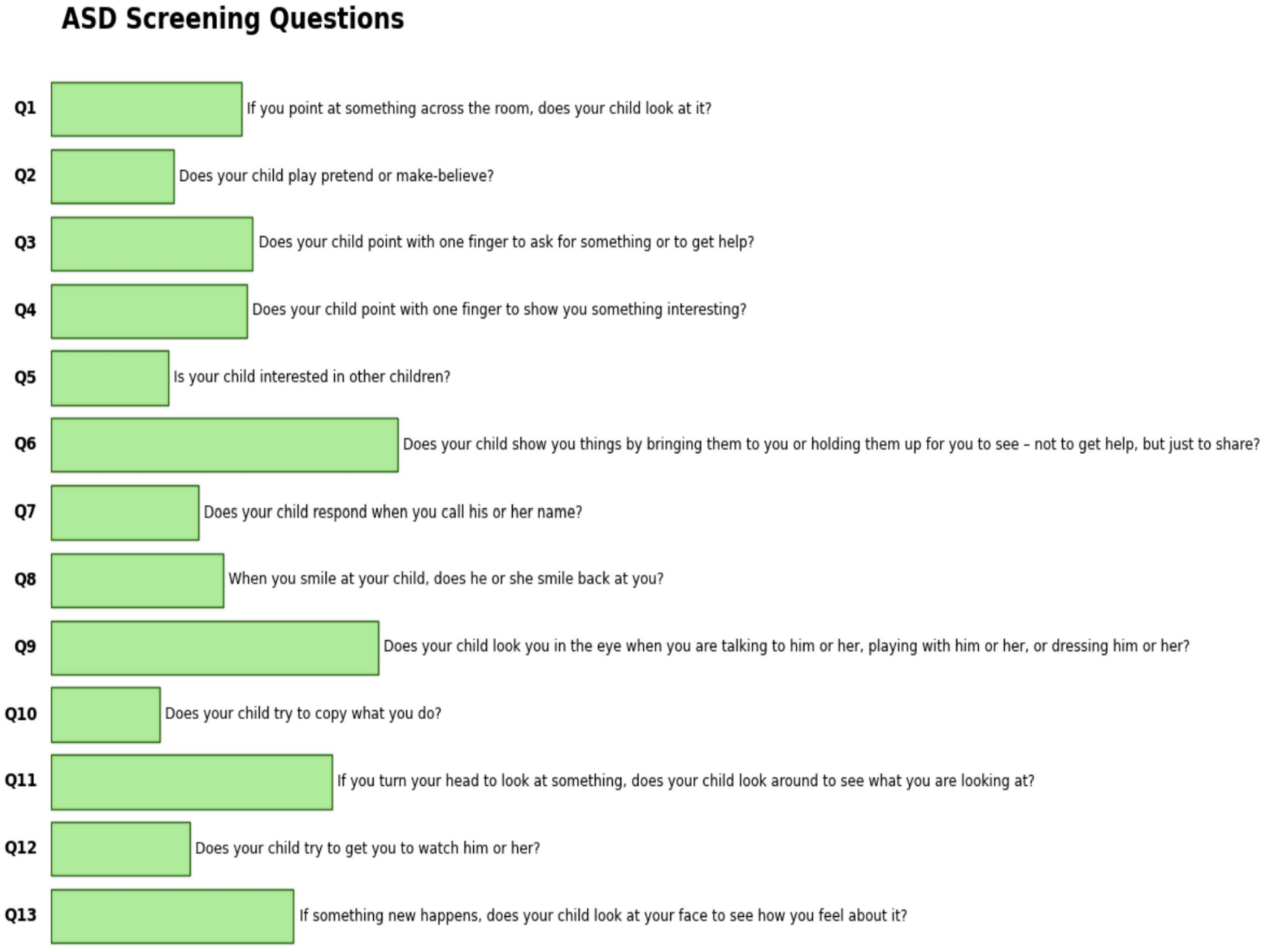
Features of the Egypt children dataset.

The dataset was obtained from the Data Science Bank repository and was constructed using the M-CHAT-R test, which comprises 13 questions. The dataset was collected from North Cairo Governorate, Egypt, with participants aged between 3 and 15 years. This dataset was collected through social media, in accordance with the questions shown in Figure 4.

### 3.2 Data acquisition

The Data acquisition prepares the data to import the dataset into a Data Frame and then examines the structure of its columns. Label encoding converts all category text attributes into numerical values. The ML algorithms can be utilized effectively to analyze and detect ASD. The Standard Scaler method is used to normalize data to a mean of zero and a variance of one, which makes the feature set more comparable in scale.

#### 3.2.1 Analyzing features of the Saudi Arabia dataset

**Figure 5:**
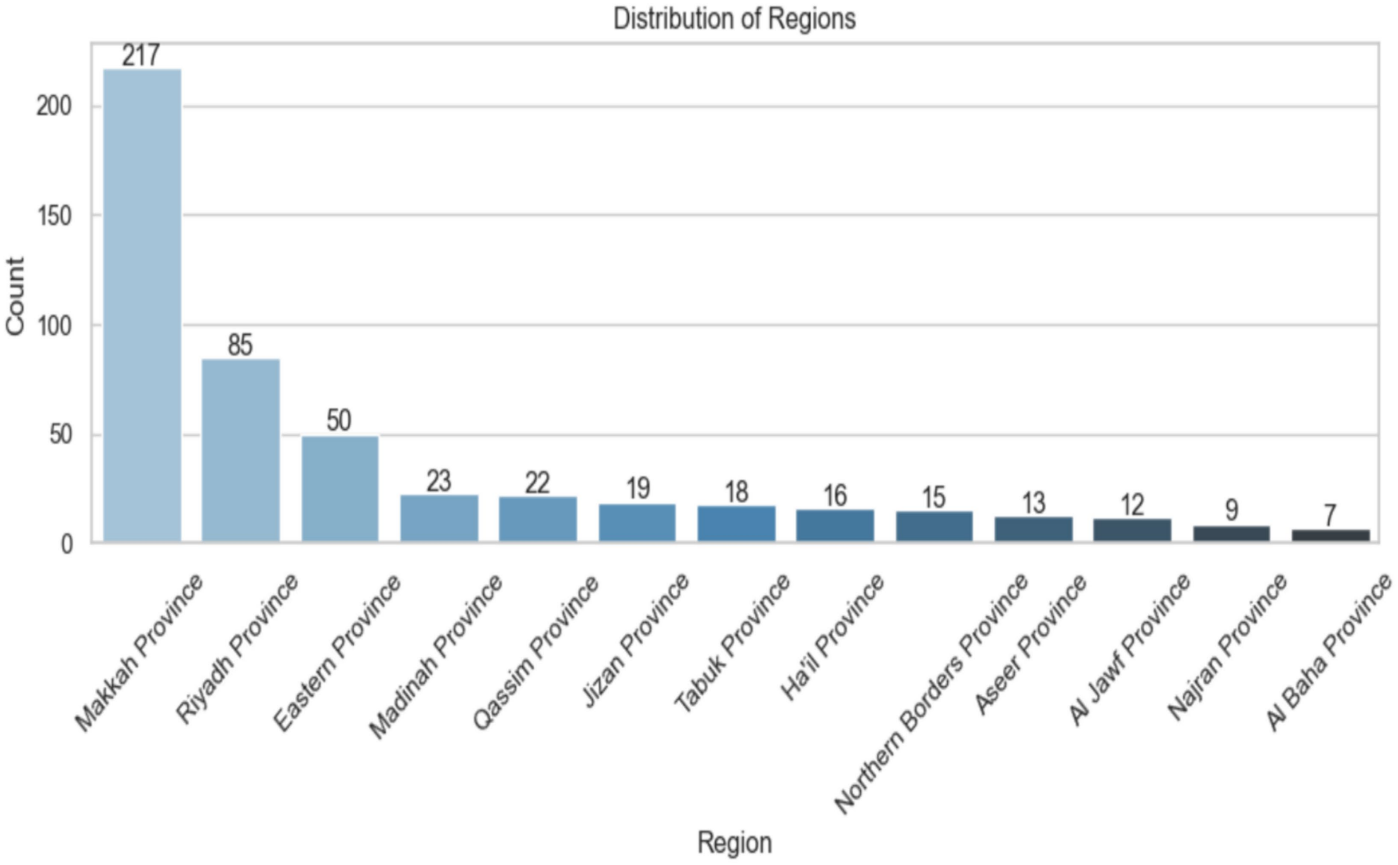
Distribution region of Saudi Arabia.

Figure 5 shows the distribution of data samples across different provinces in Saudi Arabia. The x-axis lists the province names, while the y-axis indicates the number of samples from each. There are 217 samples from Makkah Province, 85 from Riyadh Province, and 50 from Eastern Province. The other provinces have fewer samples, with Al Baha Province having the least at only 7. The bars are color-coded from light to dark blue, showing a decrease in the number of samples. Each bar also displays its exact count for clarity. The image clearly highlights the regional data imbalance immediately.

The data presented in Table 1 show that the average age of female participants was 24.67 years (SD = 8.42), while the average age of male participants was 24.03 years (SD = 8.15). Participants of all genders, aged 13 to 38, were included, with a median age of 23.0 years for each group. These two distributions are very similar, indicating that there is no significant age difference between men and women in the sample.

**Table 1:** Regional distribution of the collecting ASD dataset.

Gender	Mean	Std	Min	Max	Account	50%
Female	24.67	8.42	12.0	36.0	348.0	24.0
Male	24.03	8.15	12.0	36.0	157.0	24.0

**Figure 6:**
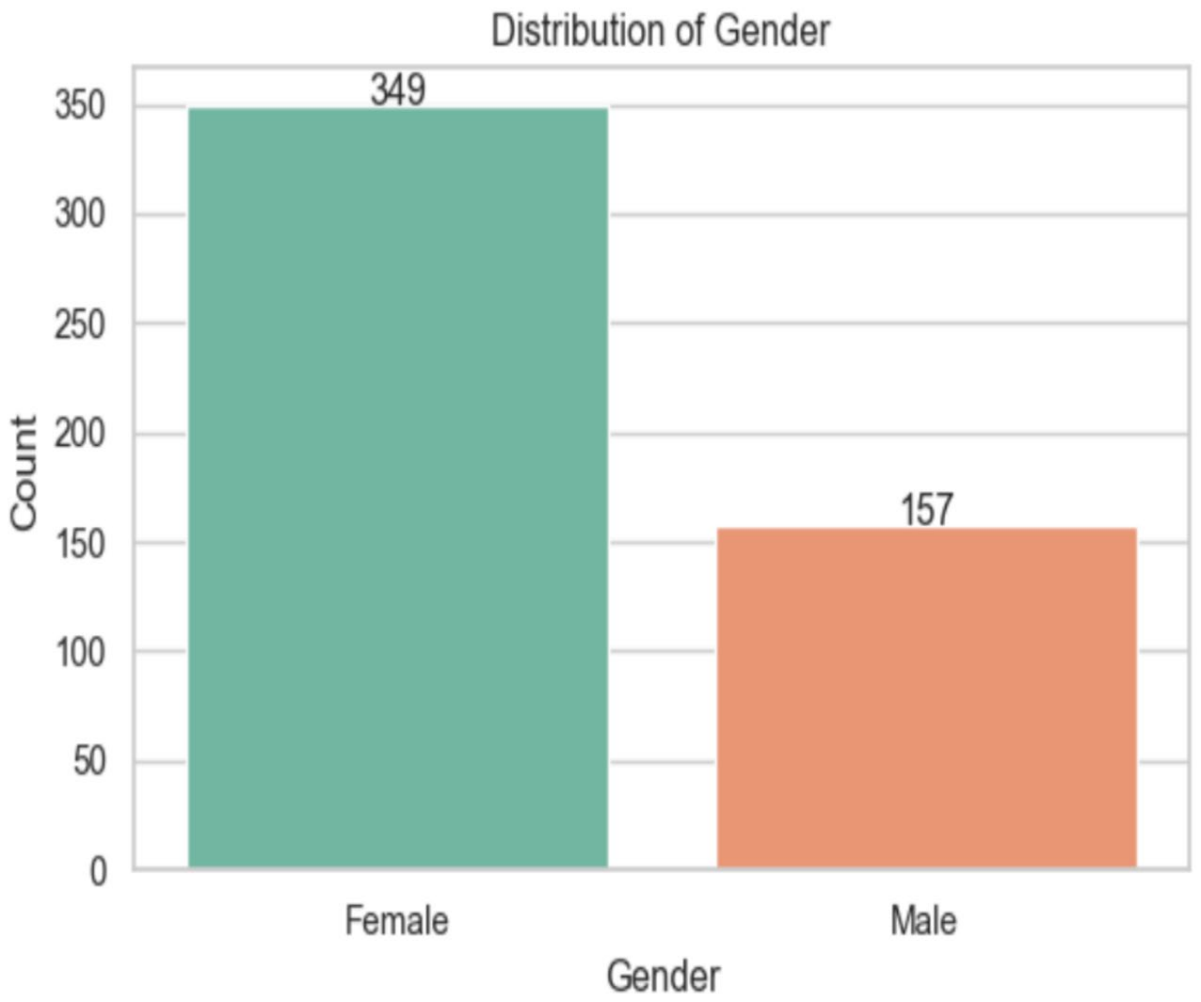
Analysis of the Gender of the dataset.

Figure 6 illustrates the ASD distribution in the KSA region. It shows that there are more females (349) than males (157) in the dataset, and the average age for both groups is nearly the same (females: 24.67 years, males: 24.03). Both groups are between 12 and 36 months old. The middle 50% of females are aged 18 to 33, while the middle 50% of men are aged 17 to 32.

**Figure 7:**
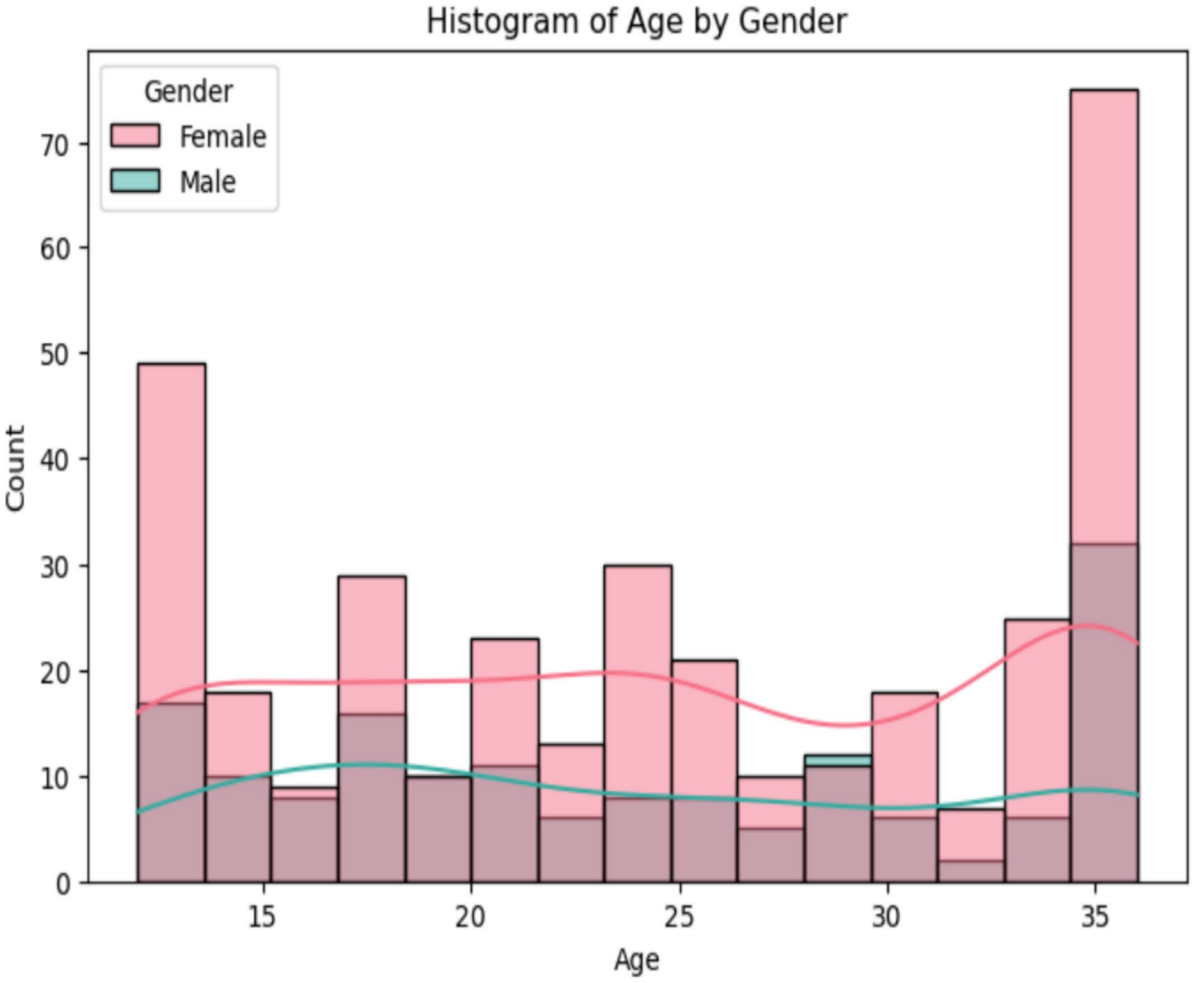
Analysis age of the dataset.

The analysis of age distribution by gender reveals that women outnumber men, as shown in Figure 7. The ages of about 12 and 35–36 months are the extremes of the age range. Between ages 15 and 30, the gender gap decreases somewhat, as females consistently outnumber males. Analyzing the density curves reveals that the age distribution among women is more even. Furthermore, men’s density is lower overall, with smaller peaks, indicating a smaller male population in each age group.

**Figure 8:**
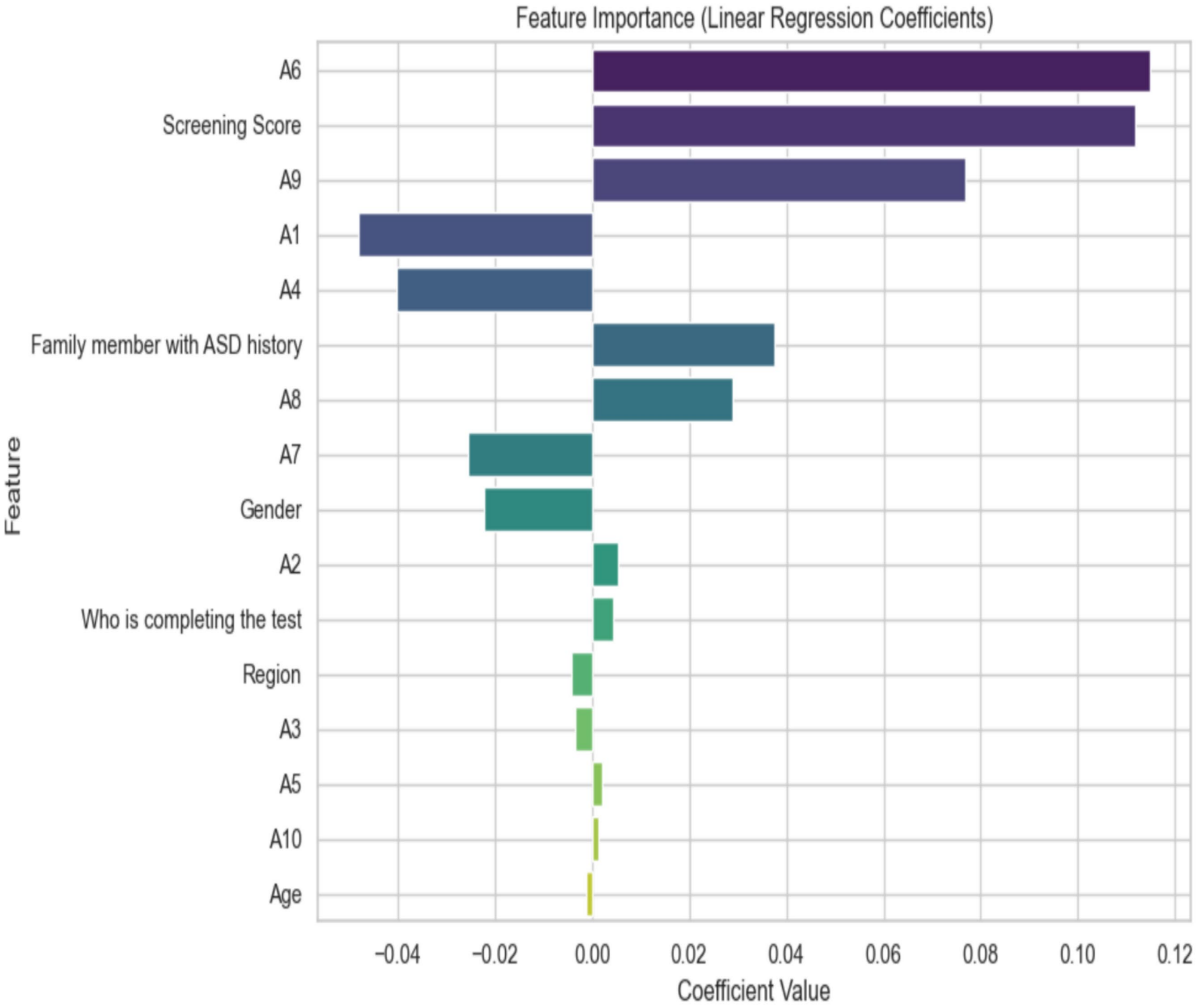
Important features using linear regression.

The importance of distinct features in predicting a result using the coefficients of the individual components, which are likely related to ASD, is shown in Figure 8. The [Screening Score (A6)] is the most important predictor, followed closely by [Family member with ASD history (A4)] and (A1). On the other hand, [Age (A0)] and [Region (A5)] have not much of an effect, since their coefficients are very close to zero. This illustrates the importance of targeting interventions at the most significant variables.

**Figure 9:**
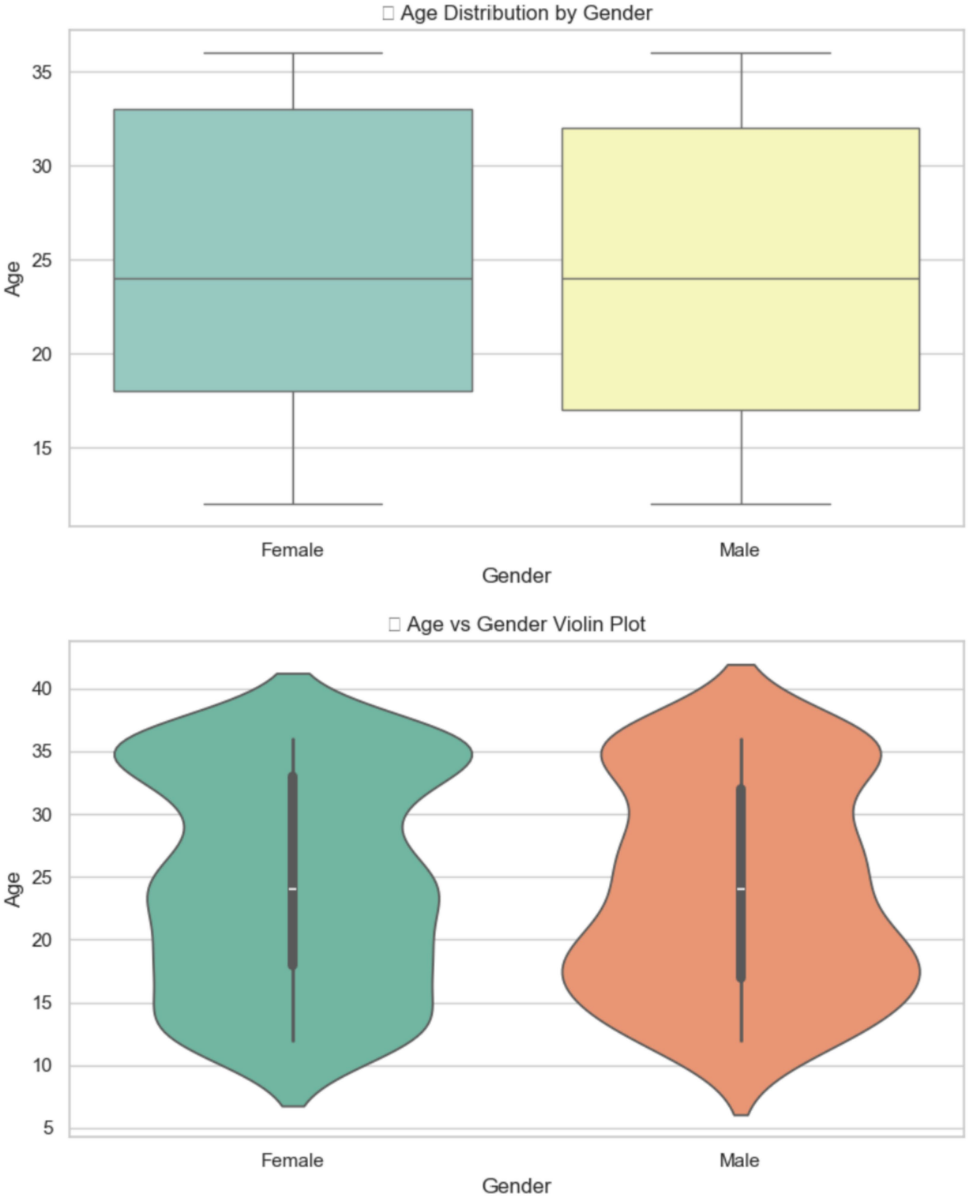
The relation between age and gender.

The differences in age distribution between men and women are shown in Figure 9. The plots reveal that all genders have a median age of about 30 months, and their interquartile ranges are quite similar. However, the age range for men is slightly broader. This suggests that the age distribution for all genders is roughly symmetrical, but males exhibit a wider range, indicating greater age variability. Overall, these ages do not vary significantly between genders, but the age range and distribution patterns do, especially among males.

#### 3.2.2 Data acquisition Egypt children of ASD dataset

**Figure 10:**
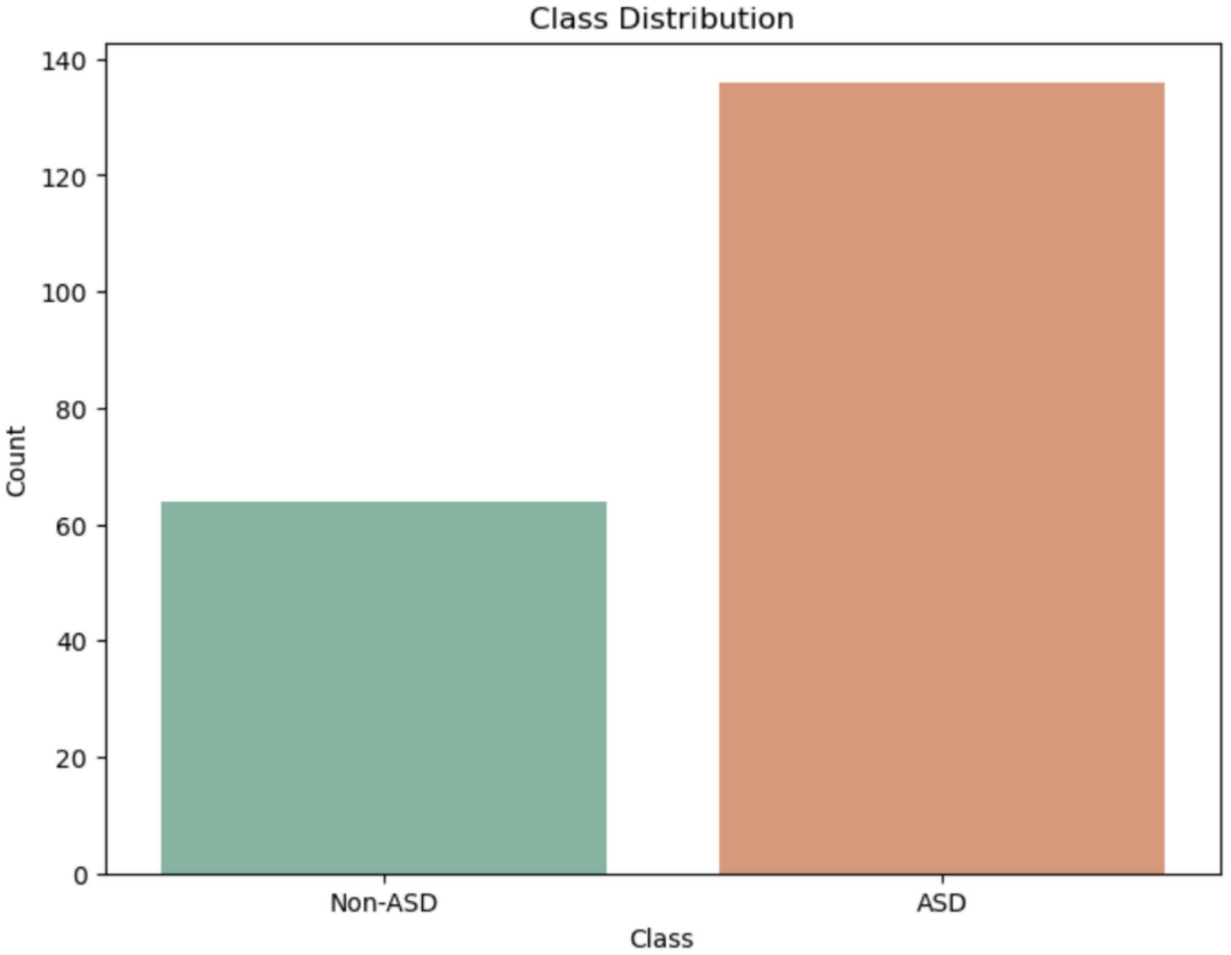
Class of Egypt children of ASD dataset.

Figure 10 shows the class distribution of Egyptian children in the ASD dataset. The orange color indicates the ASD class, which has a value of over 136, while the green color for Non-ASD shows a value just under 64.

**Figure 11:**
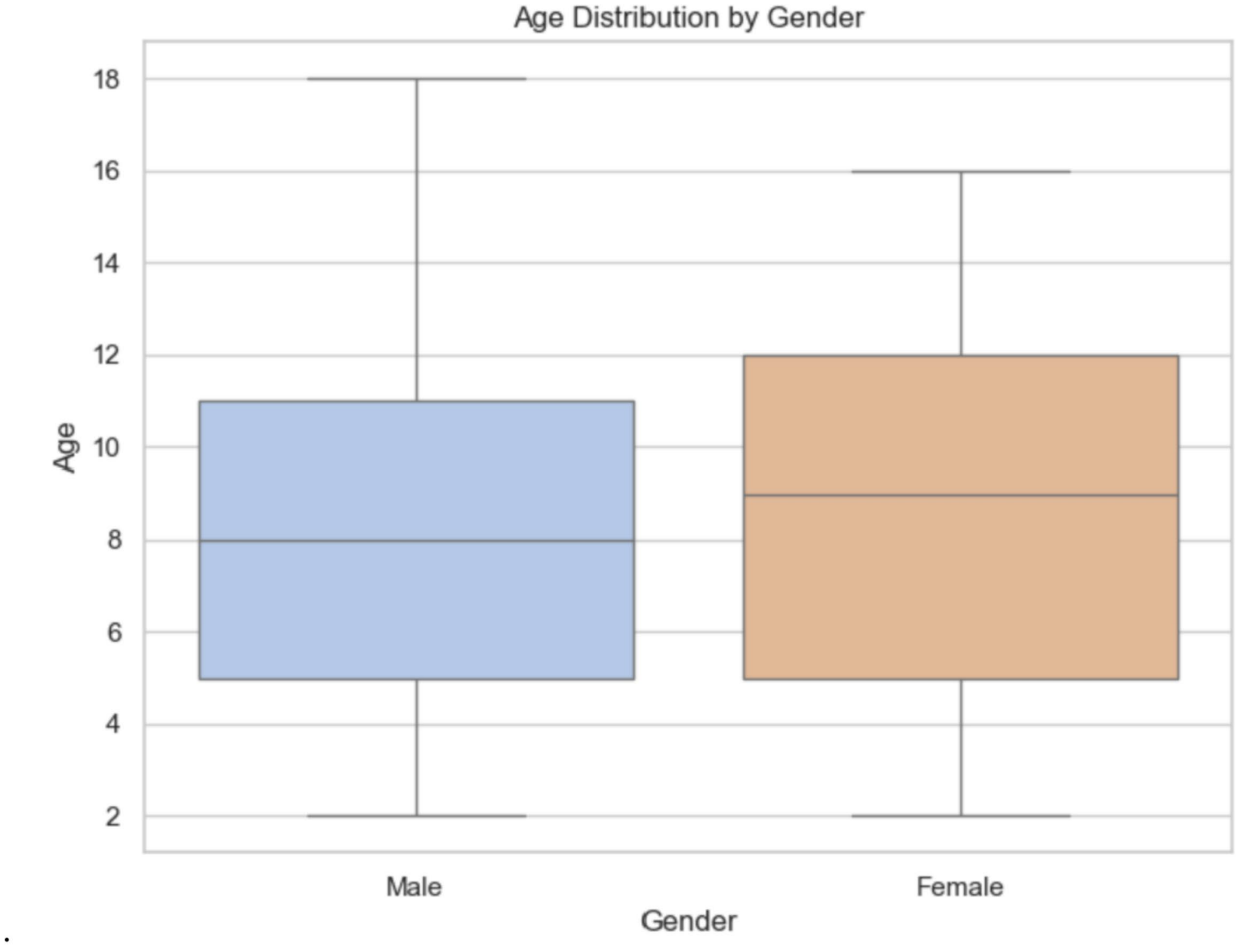
Gender distribution in the Egypt dataset.

The boxplot 11 illustrates the gender distribution, showing the number of males and females by age. Males range from 2 to 18 years old, with a median age of 8 and an interquartile range (IQR) of 5 to 11 years. Females’ ages also span from about 2 to 16 years, with an IQR of approximately 5 to 12 years and a median age of about 9, slightly higher than that of males (Figure 11).

### 3.3 Machine learning methods

#### 3.3.1 Decision tree

The DT model employs recursive splitting of the dataset into smaller sections based on the most important features that minimize uncertainty in predicting the target variable (ASD vs. Non-ASD). The DT algorithm determines the feature and threshold that best distinguish the classes, typically by maximizing impurity reduction using the Gini Index. The tree structure consists of internal nodes representing decision rules, branches showing the results of those rules, and leaf nodes indicating the predicted class. During prediction, a new sample navigates through the tree by following the decision rules at each node until it reaches a leaf node, where it receives the calculation of DT is shown in Equations 1–3.


$$\text{Entropy}=(A)=\sum_{i=1}^{C}p_{i}\;\log_{2}\;p_{i}$$

Where the ASD and non-ASD in the training dataset, the number of a class in the dataset labeled as ASD and non-ASD. The probability of the class in the training/testing dataset/testing dataset.


$$\text{entropy}\;(A\;|B)=\sum_{j=1}^{j}\frac{|s_{i}|}{|S_{i}|}\;\text{entropy}\;(A_{i})$$ (2)

Where the splitting dataset is indicated in B , S is the total the dataset, $S_{i}$ is subset of BandS and entropy(A∣B) is entropy of features.


$${\text{Gain}}^{\left(A|B\right)=entropy\left(A\right)-entropy\left(A|B\right)}$$ (3)

Where Gain (A∣B) is ASD after splitting the dataset, entropy(A) is values of entropy before dividing the decision tree.

#### 3.3.2 K-nearest neighbors (KNN)

The KNN method predicts data points based on their proximity to each other. It is a widely used and easy-to-understand classifier in ML for both classification and regression. Calculating the distance between a query point and all other data points is necessary to find the nearest neighbors. When inputting ASD, the KNN algorithm uses the Euclidean distance method to calculate the distance between it and all training samples. It then selects the *k* closest neighbors and classifies the input based on the most common class among them. We have used *k* = 5 nearest neighbors, labeled “ASD,” to classify the input as ASD. To ensure that no single characteristic dominates the distance calculation, and since KNN relies on its data, scaling is crucial. The Euclidean distance is shown in Equation 4.


$$N_{i}=\sqrt{(a_{1}-a_{2})+{\left(b_{1}-b_{2}\right)}^{2}}$$ (4)

Where a_1_, a_2_, b_1_, and b_2_ are features of the ASD dataset.

#### 3.3.3 Xplainable AI (XAI)

XAI methods are becoming increasingly important in ML to enhance accountability, trust, and transparency in complex models. Traditional XAI approaches applied to black-box models (like deep learning and ML algorithms such as RF and XGBoost) are often hesitant to adopt complicated models because they hide the decision-making process. XAI helps researchers understand, interpret, and trust the results produced by these models. Recent advances include the development of replacement models that mimic complex algorithms while remaining interpretable, as well as feature attribution techniques and rule-based explanations. These methods clarify predictions and reveal potential weaknesses in the models. Using PFI, LIME, and SHAP, we provide a detailed explanation of our ML models. PFI offers feature rankings based on their impact on performance. LIME adds local insights to help clinicians identify which features are most important for specific diagnoses. SHAP enhances our understanding of model behavior at both the global and local levels through detailed explanations.

## 4 Experiment

In this section, we have analyzed the ASD dataset by using ML, namely DT and KNN models, for detecting and diagnosing ASD. The models were methodically measured using various performance metrics. Methods were applied to enhance the clarity and understanding of the models. This study used 5-fold cross-validation. The implementation was carried out in Python programming.

### 4.1 Evaluation metrics

The evaluation metrics that employed to diagnosing the ASD system is displayed Equations 5–12.


$$\mathrm{MSE}=\frac{1}{n}\;\sum_{i=1}^{n}{\left(y_{i,\text{obser}}-y_{i,\text{pred}}\;\right)}^{2}$$ (5)

$$\text{RMSE}=\sqrt{\sum_{i=1}^{n}\frac{{\left(y_{i,\text{obser}}-y_{i,\text{pred}}\right)}^{2}}{n}}$$ (6)

$$R^{2}=\mathrm{bn}1-\frac{\sum_{i=1}^{n}\;{\left(y_{i,\text{obser}}-y_{i,\text{pred}}\right)}^{2}}{\;\sum_{i=1}^{n}\;{\left(y_{i,\text{obser}}-y_{\mathrm{avg},\exp}\right)}^{2}\;}$$ (7)

$$\text{Accuracy}=\frac{\mathrm{TP}+\mathrm{TN}}{\mathrm{TP}+\mathrm{FP}+\mathrm{FN}+\mathrm{TN}}\times 100\%$$ (8)

$$\text{Sensitivity}=\frac{\mathrm{TP}}{\mathrm{TP}+\mathrm{FN}}\times 100\%$$ (9)

$$\text{Precision}=\frac{\mathrm{TP}}{\mathrm{TP}+\mathrm{FP}}\times 100\%$$ (10)

$$\text{Fscore}=\frac{2*\text{preision}*\text{Sensitivity}}{\text{preision}+\text{Sensitivity}}\times 100\%$$ (11)

$$R\%=\frac{n(\sum_{i=1}^{n}y_{i,\text{obser}}\times y_{i,\text{pred}})-(\sum_{i=1}^{n}y_{i,\text{obser}})(\sum_{i=1}^{n}y_{i,\text{pred}})}{\sqrt{\left[n{\left(\sum_{i=1}^{n}y_{i,\text{obser}}\right)}^{2}-{\left(\sum_{i=1}^{n}y_{i,\text{obser}}\right)}^{2}][n{\left(\sum_{i=1}^{n}y_{i,\text{pred}}\right)}^{2}-{\left(\sum_{i=1}^{n}y_{i,\text{pred}}\right)}^{2}\right]}}\times 100$$ (12)

Where $y_{i,\text{obser}}$ is a testing dataset, $y_{i,\text{pred}}$ is the output of the ML model from testing the dataset, TP- true positive, FP- false positive, FN false negative, TN true negative.

### 4.2 XAI results

#### 4.2.1 XAI results of ADS Saudi Arabia dataset

**Figure 12:**
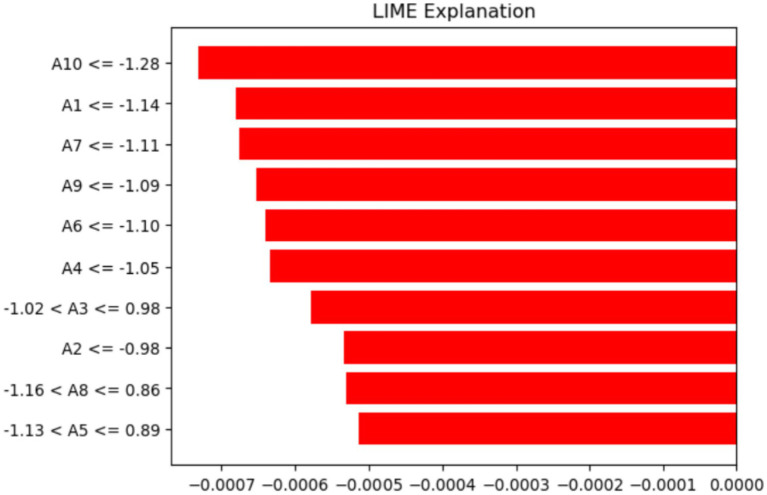
Analysis of features using the LIME method.

The LIME method helps improve ML model predictions by showing how different features influence specific traits. Figure 12 displays the features that made it harder for the ML model to analyze and identify ASD using LIME. The pattern indicates that particular threshold requirements for several attributes (A1–A10) are crucial, as values within these thresholds have a major impact. These insights deepen our understanding of the features that lower the chances of the model recognizing an individual with ASD.

The labels on the y-axis show the features and the conditions they meet for the data instance being discussed. For example (A10–1.28), indicates that feature A10’s value is less than or equal to −1.28. Similarly, (−1.02 < A3 ≤ 0.98) shows that A3’s value is between −1.02 and 0.98. Lastly, (−1.13 < A5 ≤ 0.89) suggests that A5 falls within that range. These conditions match the discretized feature values that LIME uses to approximate the model’s local behavior and highlight the feature ranges that most significantly influence the prediction.

**Figure 13:**
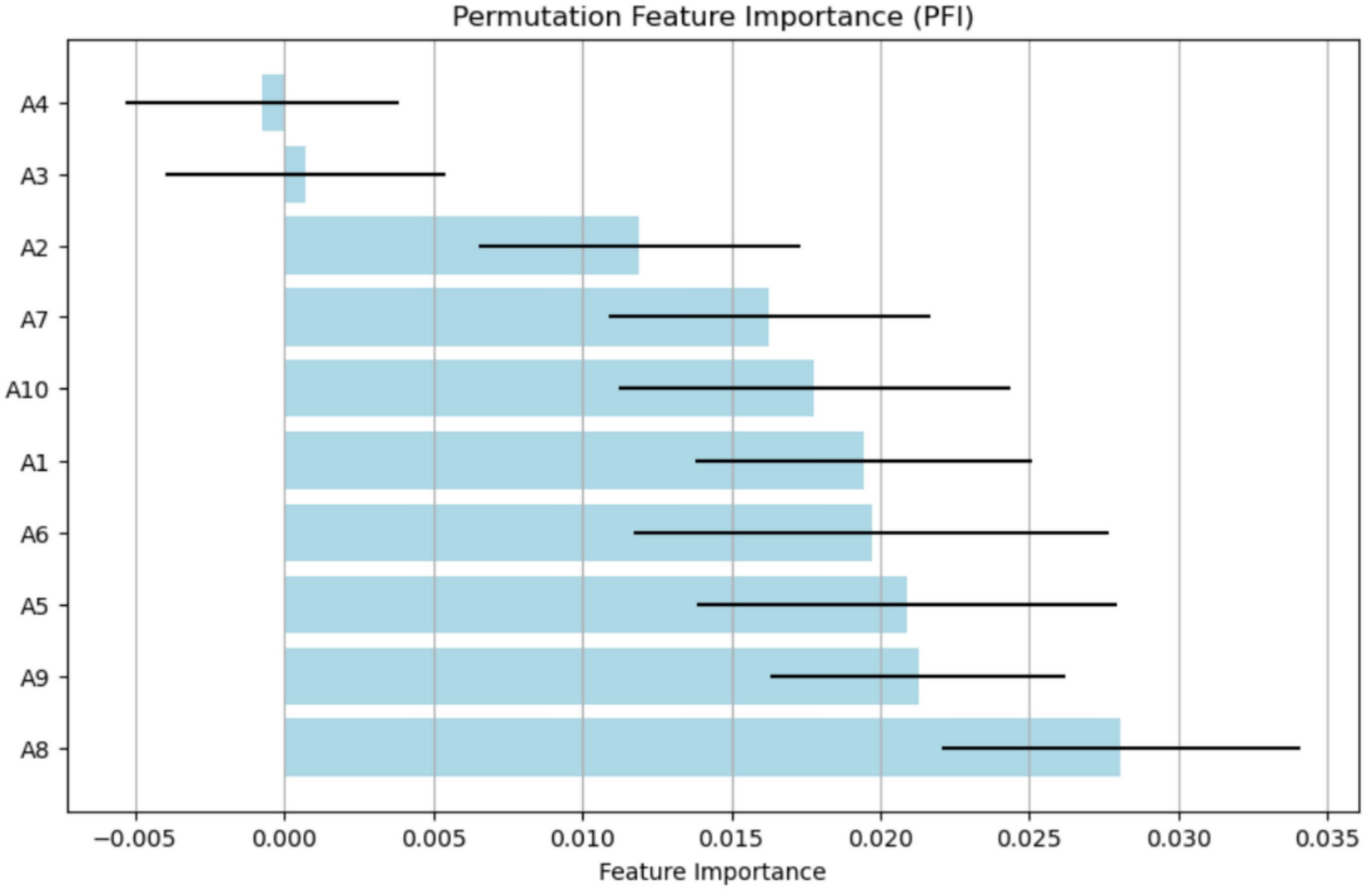
Analysis of features using the PFI method.

The PFI method identifies the features that most significantly affect the ASD model’s performance when their values are randomly permuted, as shown in Figure 13. The x-axis displays the (Feature Importance) values, ranging from about −0.005 to 0.035. The y-axis lists different attributes (A1, A2, A3, etc.), indicating their importance within the model. A8 stands out with its high importance, highlighting its crucial role in accurate prediction. The features A9, A5, and A6 also contribute notably. Additionally, A3 and A4 have minimal or slightly negative importance, suggesting they have little to no predictive value and may introduce noise. This study finds A8 and A1 to be the most relevant features, while A3 and A4 are the least influential for the model.

**Figure 14:**
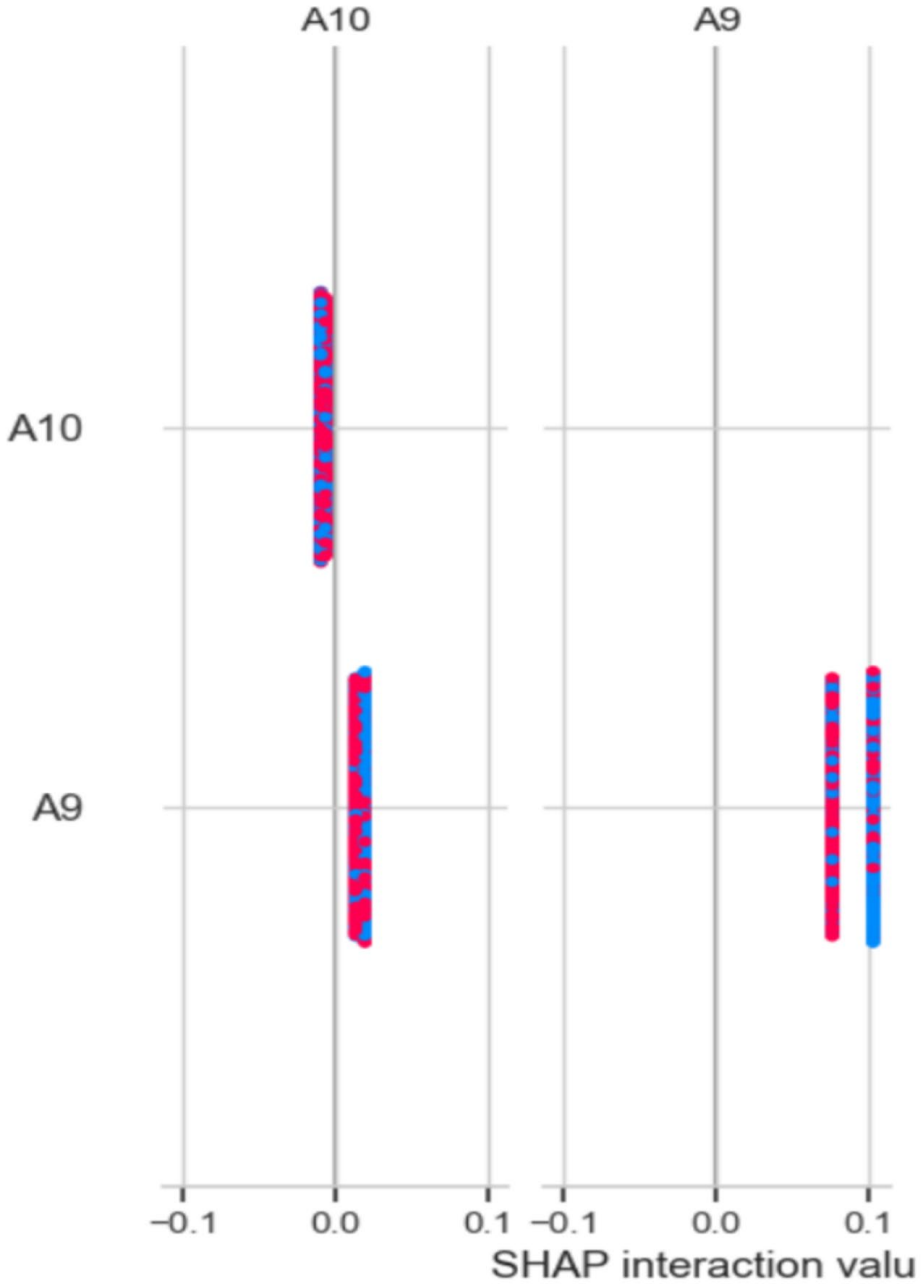
Analysis of the feature using the SHAP method.

**Figure 15:**
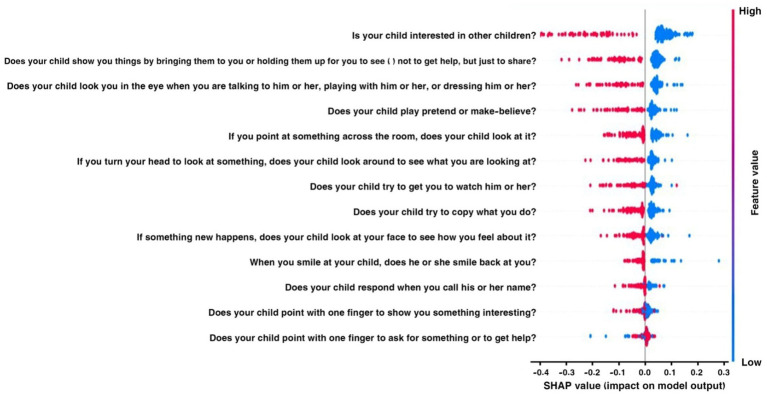
SHAP results of the Egypt ASD dataset.

Figure 14 shows the results of the SHAP model for identifying important features. The x-axis displays SHAP interaction values, which range from −0.1 to 0.1. A9 and A10 are the two features listed on the y-axis. Data points for each feature are shown as colored markers, representing different categories or conditions. The spread of these points illustrates how SHAP interaction values vary across different features, highlighting the interactions and their impact on the model’s predictions. The clustering of points around certain values suggests that the interactions between characteristics have different levels of strength (Figure 15).

#### 4.2.2 XAI results of the ADS Egypt dataset

The SHAP plot 15 illustrates how various traits affect the model’s output related to behaviors associated with ASD in children. The SHAP values, which show the influence of each feature on the model’s predictions, are displayed on the horizontal axis. The features are listed along the vertical axis.

Positive SHAP values indicate that the model is more likely to predict ASD when certain behavior scores are higher. Conversely, negative values suggest the opposite. Questions like “Does your child show you things by bringing them to you?” and “Does your child look you in the eye when talking?” carry very positive implications, and low scores on these items would strongly indicate ASD. Conversely, questions with negative values, such as “Does your child point with one finger to ask for something interesting?” suggest that the likelihood of an ASD diagnosis decreases as the child engages more in this behavior. This visual tool effectively highlights important behavioral markers that influence the model’s predictions, making it easier to understand the behavioral traits associated with ASD.

**Figure 16:**
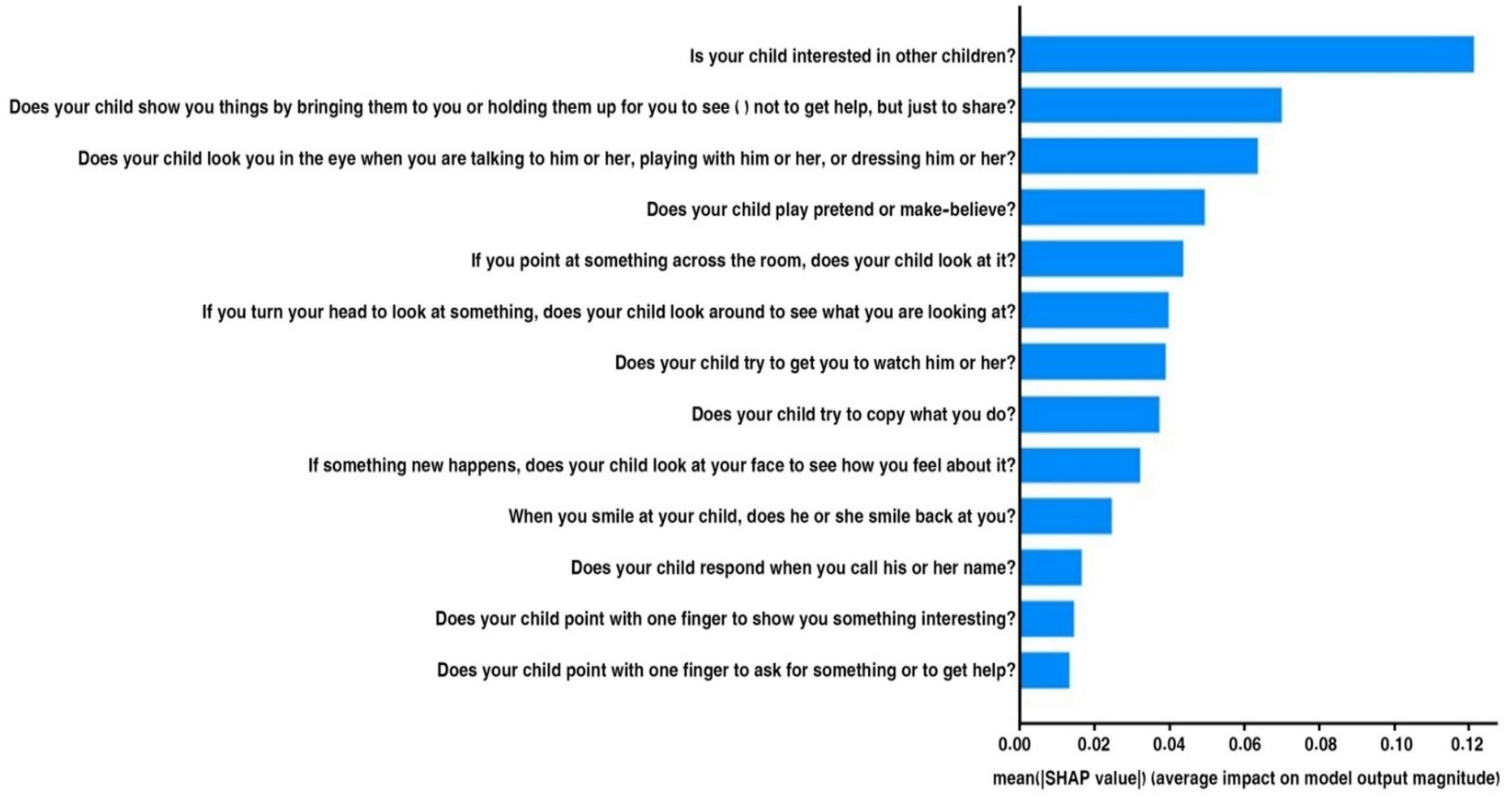
Mean SHAP of the Egyptian dataset.

Figure 16 illustrates the mean absolute SHAP values for several factors that influence the model’s predictions regarding behaviors associated with ASD in children. The vertical axis displays the features one at a time, and the horizontal axis indicates the average impact of each feature on the model’s output size.

“Is your child interested in other children?” was the most important characteristic for predicting ASD, followed by the others in order of importance. Other important behaviors linked to ASD include: “Does your child show you things by bringing them to you?” and “Does your child look you in the eye when talking?” It seems that not being involved in these areas is a strong sign of ASD. The lower average effects of traits such as “Does your child point with one finger to ask for something interesting?” suggest that some behaviors may not be as indicative of ASD. By examining the most significant behavioral signs employed by the model, we can gain a deeper understanding of the traits associated with ASD through this visual representation.

In this research, the gap was addressed by incorporating SHAP into clinical data for the diagnosis of ASD. Official health doctors can use SHAP values to identify environmental factors most likely to cause autism in each child, enabling more personalized treatments. Furthermore, this study explores how the LIME model can help teachers by providing a clearer understanding of complex model predictions during class or counseling sessions. The research can also assist policymakers in using tree-based models to rank the importance of features for community-level preventive actions against environmental risk factors.

### 4.3 Machine learning results of the Saudi Arabia dataset

#### 4.3.1 . KNN results

Table 2 shows how effective KNN is at detecting and analyzing ASD in Saudi Arabia. The KNN model achieves an overall accuracy of 97%, demonstrating a reliable classification ability. In the non-ASD group, the model achieves 93% accuracy, 98% recall, and a 95% F1 score, demonstrating its ability to correctly identify individuals without ASD with high confidence and a low false-negative rate. In the ASD group, accuracy is 99%, with a 96% recall and a 97% F1 score, demonstrating the model’s strong ability to detect ASD cases while balancing precision and recall. The macro average scores, consistently at 97%, reflect steady performance across both groups.

**Table 2:** Results of the KNN.

Class name	Precision (%)	Recall (%)	F1 score (%)	Support
Non-ASD	93	98	95	165
ASD	99	96	97	341
Accuracy		97		
Macro Avg	96	97	96	

**Figure 17:**
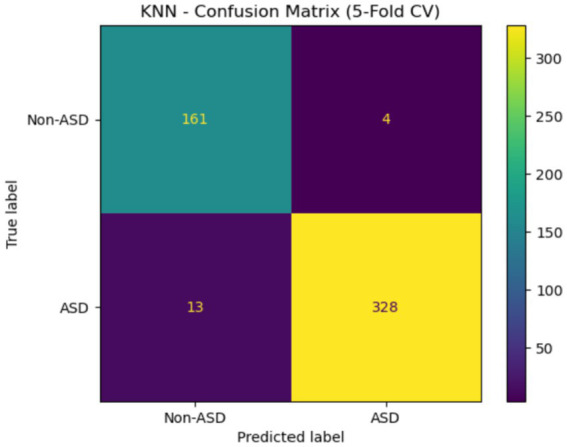
Confusion matrix of KNN of the Saudi Arabia dataset.

Figure 17 displays the confusion matrix for the KNN model with 5-fold cross-validation. The model accurately classified 161 Non-ASD and 328 ASD cases, with only 4 Non-ASD instances misclassified as ASD and 13 ASD cases misclassified as Non-ASD. The results demonstrate high sensitivity and specificity, emphasizing the model’s effectiveness in detecting ASD with minimal errors.

#### 4.3.2 Decision tree

Table 3 shows that the DT model performs strongly in detecting and analyzing ASD in Saudi Arabia, with an overall accuracy of 95%. The DT model has a precision of 92%, a recall of 92%, and an F1 score of 92% for the Non-ASD class, indicating effective identification of individuals without ASD with balanced sensitivity and specificity. For the ASD class, the DT model demonstrates improved performance metrics, achieving a precision of 96%, a recall of 96%, and an F1 score of 96%. These results highlight its success in accurately identifying ASD cases while reducing false negatives. The macro average of DT scores across all metrics is 94%.

**Table 3:** Results of the DT.

Class name	Precision (%)	Recall (%)	F1 score (%)	Support
Non-ASD	92	92	92	165
ASD	96	96	96	341
Accuracy		95		
Macro Avg	94	94	94	

**Figure 18:**
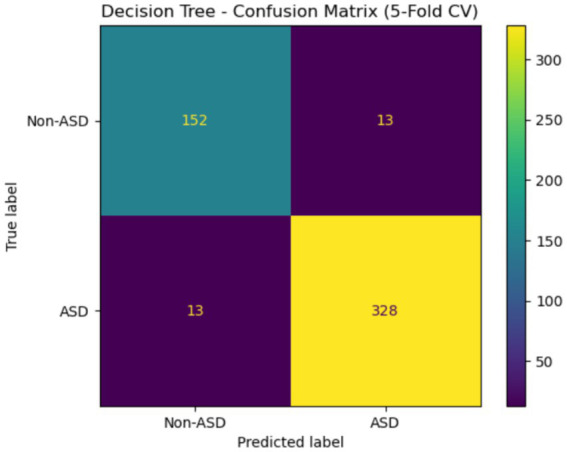
Confusion matrix of DT of the Saudi Arabia dataset.

Figure 18 shows the confusion matrix for a DT model evaluated with 5-fold cross-validation. The model correctly classified 152 non-ASD instances and 328 ASD instances, while it misclassified 13 non-ASD cases as ASD and 13 ASD cases as non-ASD. This indicates high accuracy in predictions, especially for the ASD class, but also highlights challenges in distinguishing between the two categories, as evidenced by equal misclassification rates in both directions.

### 4.4 Machine learning results of the Egyptian children of ASD dataset

#### 4.4.1 KNN results

Table 4 demonstrates the effectiveness of the KNN model in classifying ASD. The model attained 85% Precision, 89% Recall, and 87% F1-score for the non-ASD class. For the ASD class, it achieved 95% Precision, 93% Recall, and 94% F1-score. The model had a Macro-Averaged Precision of 90%, a Recall of 91%, and an F1-score of 90%. It effectively distinguished between cases with and without ASD, with an overall accuracy of 92%.

**Table 4:** Results of the KNN.

Class name	Precision (%)	Recall (%)	F1 score (%)	Support
Non-ASD	85	89	87	64
ASD	95	93	94	136
Accuracy		92		
Macro Avg	90	91	90	

**Figure 19:**
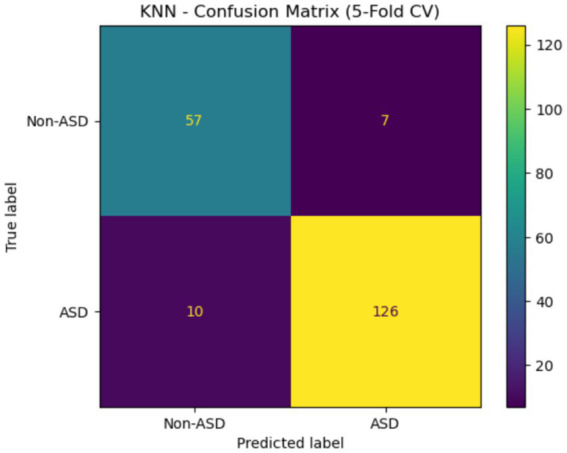
Confusion matrix of the KNN Egypt ASD dataset.

The confusion matrix for KNN is shown in Figure 19. This assessment is based on a 5-fold cross-validation technique. The four quadrants are: The top left (57) represents true negatives, which are cases that do not have ASD. The top right (7) shows FP, or cases that are not ASD but are incorrectly labeled as such. The bottom left (10) indicates actual ASD cases that were mistakenly classified as non-ASD. Lastly, the bottom right (126) displays TP, which are cases correctly identified as ASD. This matrix demonstrates that the model can effectively detect ASD, as evidenced by a high TP rate.

#### 4.4.2 DT results

Table 5 presents the results of the DT model on the Egyptian children’s ASD dataset. The model achieved an F1-score of 81%, a recall of 84%, and a precision of 78% for the Non-ASD class, with a recall of 89%, an F1-score of 91%, and an accuracy of 92%. The model was 88% accurate overall, with a recall of 87%, an F1-score of 86%, and a precision of 85%. These results show that the model is effective, as it more accurately identifies ASD cases than Non-ASD cases by a small margin.

**Table 5:** Results of the DT.

Class name	Precision (%)	Recall (%)	F1 score (%)	Support
Non-ASD	78	84	81	64
ASD	92	89	91	163
Accuracy		88		
Macro Avg	85	87	86	

**Figure 20:**
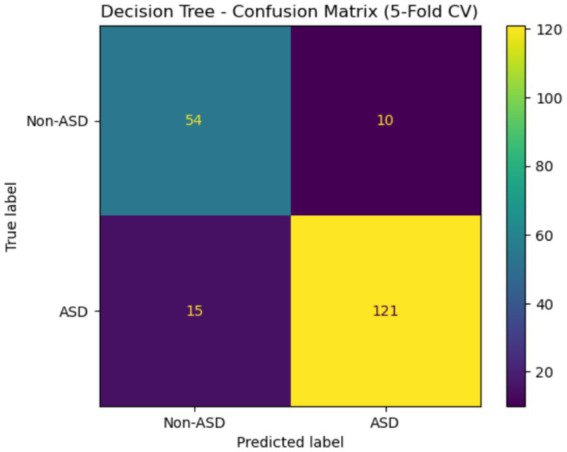
Confusion matrix of DT Egypt ASD dataset.

Figure 20 shows the confusion matrix for the DT classifier that distinguished between instances with and without ASD when tested with 5-fold cross-validation. The accuracy rate for cases not related to ASD was 54, with 10 of those cases incorrectly identified as ASD. There were 121 actual cases of ASD, but 15 of them were wrongly labeled as non-ASD. Although there are still a small number of misclassifications for both classes, the model performs well overall and is very effective at identifying patients with ASD.

### 4.5 Statistical analysis of the Saudi Arabia dataset

The results of the KNN analysis are presented in Table 6. The KNN model demonstrates that different models and variables show varying performance levels based on the evaluation criteria. The prediction error for KNN is indicated by a low MSE of 0.0335, a very high RMSE of 0.1801, and a substantial R2 value of 84.72%. In comparison, the decision tree has lower prediction errors, with an MSE of 0.0514 and a smaller RMSE of 0.2261, along with a lower R2 of 76.61%. These results highlight disparities in feature-specific variance explanation and predictive accuracy, indicating that the KNN model outperforms the Decision Tree in predicting outcomes within the ASD dataset.

**Table 6:** Model analysis by using statistical metrics.

Models	MSE	RMSE	R^2^
KNN	0.0335	0.1801	0.8472
DT	0.0514	0.2261	0.7661

The Chi-square test of independence was used to examine the relationship between each questionnaire item (A1–A10) and the diagnostic category (ASD vs. Non-ASD). We created a contingency table for each item to compare how the two groups responded, then calculated the Chi-square statistic and its *p*-value. A statistically significant result (*p* < 0.05) indicates that responses to the question depend on diagnostic status, suggesting that the item helps distinguish between individuals with ASD and those without.

Table 7 shows a chi-square analysis of the ASD dataset, indicating that most questionnaire items (A1-A10) are strongly related to the target class, with *p*-values (*p* < 0.001) and chi-square values ranging from 18.30 to 233.38. This suggests that these items are very effective at predicting ASD. The highest chi-square values were observed for A6, A9, and A2, highlighting their importance in grouping. Additionally, the variable Region was found to be statistically significant (χ^2^ = 68.75, p < 0.001), implying that regional differences may significantly influence ASD prediction. In contrast, gender did not show a statistically significant link with ASD diagnosis in this sample (χ^2^ = 1.36, *p* = 0.24). These findings emphasize the importance of various questionnaire attributes and geographic factors, regardless of gender.

**Table 7:** Chi-square analysis Saudi Arabia dataset.

Question	Chi2	*p*-value	Significant
A1	142.504737	7.542565e-33	True
A2	192.034563	1.143515e-43	True
A3	147.226566	7.001691e-34	True
A4	158.252141	2.726134e-36	True
A5	148.431639	3.817488e-34	True
A6	233.380301	1.091888e-52	True
A7	138.990403	4.425832e-32	True
A8	162.219919	3.703761e-37	True
A9	215.878602	7.168092e-49	True
A10	18.303141	1.883963e-05	True
Region	68.74771416490223	5.48932140834897e-10	True
Gender	1.3632486215470982	0.24297525967385414	False

### 4.6 Statistical analysis of the Egypt dataset

Table 8 shows the statistical evaluation of the models used on the Egyptian children ASD dataset using the MSE, RMSE, and R^3^ metrics. The data suggests that the KNN and DT models had similar results. However, DT performed significantly better than KNN, as indicated by its slightly higher R^2^ score (18.13 compared to 18.09) and lower error values (MSE = 0.0374, RMSE = 0.0618). It appears that both models can comprehend the information and identify patterns, but the DT model is slightly more effective at making predictions.

**Table 8:** Model analysis by using statistical metrics of Egyptian children of ASD.

Models	MSE	RMSE	R^2^
KNN	0.038	0.0635	18.09
DT	0.0374	0.0618	18.13

The Chi-square analysis of the 13 categorical questions is shown in Table 9 of the Egyptian dataset. The (Q1, Q2, Q4–Q13) had statistically significant links to the outcome variable (*p* < 0.05). Q5 (Chi2 = 95.16, p ≈ 1.75 × 10^−22^), Q6 (Chi2 = 79.63, p ≈ 4.51 × 10^−19^), and Q9 (Chi2 = 66.69, p ≈ 3.17 × 10^−16^) showed powerful effects. The results indicate that how people answer these questions is a good way to predict the condition being studied. In contrast, Q3 did not reach statistical significance (Chi2 = 2.02, *p* = 0.15), suggesting it does not effectively differentiate between groups. The independent t-test of Age by Gender yielded a non-significant result (Chi2 = 2.70, *p* = 0.10), indicating no significant difference in age distribution between males and females in this sample.

**Table 9:** Chi-square analysis of the Egyptian dataset.

Question	Chi2	*p*-value	Significant
Q1	40.951431	1.560597e-10	True
Q2	41.520957	1.166141e-10	True
Q3	2.023391	1.548929e-01	False
Q4	12.030110	5.234799e-04	True
Q5	95.164199	1.752308e-22	True
Q6	79.632353	4.509800e-19	True
Q7	18.375892	1.813378e-05	True
Q8	8.731839	3.127009e-03	True
Q9	66.694323	3.170468e-16	True
Q10	37.226338	1.051835e-09	True
Q11	43.273043	4.760977e-11	True
Q12	44.813418	2.167324e-11	True
Q13	33.684393	6.481865e-09	True
Age	2.7048198478655157	0.1000453828138666	False

## 5 Discussion

The primary objective of this study is to enhance health sector services, thereby benefiting individuals with ASD in Arabic society. This goal is to lower stigma, give families more control, and make sure that people with autism have the same chances as everyone else to engage in society fully.

This study aimed to identify the requirements for using AI to diagnose ASD and the challenges faced by experts in KSA and Egypt. The findings not only provided valuable insights that expanded the scope of previous related research but also emphasized the significance of AI in ASD diagnosis.

The dataset was obtained from the Kaggle library, including data on children aged 12 to 36 months from multiple regions of KSA. The analysis of average age shows no significant difference in mean age between genders, highlighting accurate demographic representation in the sample. The study of answer distribution reveals trends in participant responses across various questions, with answers categorized in a binary manner. Makkah Province has the highest number of responses, with 217, followed by Riyadh Province with 85. Other areas, such as Eastern Province and Madinah Province, have much lower response counts, with 50 and 23 participants, respectively. This distribution may be due to demographic variables, access to ASD screening programs, regional knowledge of the disorder, or a family history of ASD among those who answered. About 75.9% of the people who responded said that no one in their family had ASD, while 24.1% said that at least one family member had been diagnosed with the disease.

The importance of linear regression coefficients was used to analyze ASD, which is evaluated using several criteria. Features A6 (Screening Score) and A9 have the highest positive coefficients, indicating a strong positive relationship with the outcome and emphasizing their significance in predicting ASD. A1 and A4 are two other key traits that also show positive associations. Features such as Age (A10) and A5 have negative coefficients, suggesting they may be negatively related to ASD diagnosis.

The data in Egypt was collected from the North Cairo Governorate. This dataset was gathered from social media. It included children of all genders (male and female), and their ages ranged from 2 to 15.

In the Saudi Arabian dataset, XAI methods, especially SHAP and LIME models, were used to improve model transparency by showing how features influence predictions. These techniques are part of interpretability strategies used in the feature selection process for studying ASD. SHAP values help explain how characteristics A9 and A10 interact and how these interactions affect model predictions. The distribution of SHAP interaction values shows different levels of interaction strength, helping to identify key feature dependencies in ASD diagnosis. LIME offers localized explanations of the machine learning models, highlighting features A1, A2, and A4 as important contributors, ranked by their explanatory power. Additionally, the PFI method provides an overall view of feature importance, with features like A2 and A10 demonstrating high significance, underscoring their crucial role in ASD assessment. These approaches allow for a detailed understanding of the most relevant elements in diagnosing ASD, thereby improving model interpretability and decision-making.

In the Egypt dataset, according to the SHAP method, the most important features are “Is your child interested in other children?” “Does your child show you things by bringing them to you or holding them up for you to see—not to get help, but just to share?” “Does your child look you in the eye when you are talking to him or her, playing with him or her, or dressing him or her?” and “Does your child play pretend or make-believe?” I included that the key question concerns social interaction and behaviors, especially how interested children are in other children, how they pay attention to each other, and how they make eye contact.

The findings indicate that the KNN and DT models achieved high accuracy in distinguishing between ASD and non-ASD (about 95–97%). This method can support the detection and analysis of ASD, enabling early diagnosis using the Saudi Arabia dataset. It can assist doctors and health officials in identifying ASD early, as it is a distinct condition that develops differently.

In this research, we used an additional ASD dataset from the Data Science Bank repository in North Cairo Governorate, Egypt, which included participants. This dataset covers two classes: ASD and non-ASD. It was observed that the KNN model achieved a high accuracy of 92%.

### 5.1 Limitations of the proposed work

The limitation of this study is that plans for subsequent research include user studies with clinicians and educators in Saudi Arabia to assess the clarity, usability, and practical significance of the generated explanations. This will ensure that our methodology surpasses mathematical interpretability, attaining genuine human-centered interpretability in clinical practice.

This study aimed to examine variables influencing ASD in Arab countries; however, the comparison was limited to datasets from Saudi Arabia and Egypt only. Other Arab nations were excluded due to the lack of standardized or readily available datasets. As a result, the findings may not fully represent the diverse factors affecting ASD across the broader Arab region. Future research should include a wider range of countries and larger, more diverse samples to improve generalizability.

## 6 Conclusion

In this research, we developed a system based on an AI algorithm for diagnosing ASD. The proposed model was tested using two ASD datasets. Our conclusions are as follows:

- This study uses a dataset collected from various regions in Saudi Arabia in 2022. The data was gathered through a Google form utilizing Q-CHAT-10 questions. A total of 506 participants, comprising both males and females, were included in the study.
- Additionally, we utilized ASD data from the Data Science Bank repository in North Cairo Governorate, Egypt, which includes 200 instances.
- To characterize our participants, we used frequencies for the categorical variables. An analysis was conducted to compare the KNN and DT algorithms, focusing on their accuracy, interpretability, and computational efficiency using Python programming.
- XAI techniques such as PFI, LIME, and SHAP were employed to improve ML models for diagnosing ASD. The analysis revealed that certain features are often emphasized in clinical evaluations
- The KNN method demonstrated superior accuracy, which is crucial for making informed clinical decisions, with a 97% accuracy rate using the Saudi Arabia dataset. In the Egyptian dataset, the KNN model achieved an accuracy of 92%.
- This finding enhances communication between healthcare professionals and AI systems, increasing confidence and practicality for detecting and diagnosing the ASD at early stages.
- The findings suggest that, in identifying children with ASD, specific behavioral characteristics, including social communication patterns, are more significant than demographic factors or family background. This conclusion reflects the complex nature of diagnosing ASD and highlights the important role that individual behavioral markers in children play.
- Developing our own datasets with different types of data like images, EEG, and clinical data is future work.

## Data Availability

The datasets presented in this study can be found in online repositories. The names of the repository/repositories and accession number(s) can be found at: the dataset used for the findings is publicly available at the public repository. Dataset: https://www.kaggle.com/datasets/asdpredictioninsaudi/asd-screening-data-for-toddlers-in-saudi-arabia, second dataset: https://www.scidb.cn/en/detail?dataSetId=0b84f15557744486a2d59366716d4f8d.
